# Change Point Estimation in Monitoring Survival Time

**DOI:** 10.1371/journal.pone.0033630

**Published:** 2012-03-16

**Authors:** Hassan Assareh, Kerrie Mengersen

**Affiliations:** 1 Discipline of Mathematical Sciences, Science and Engineering Faculty, Queensland University of Technology, Brisbane, Queensland, Australia; 2 St Andrew's Medical Institute, Brisbane, Queensland, Australia; UCL Institute of Child Health, University College London, United Kingdom

## Abstract

Precise identification of the time when a change in a hospital outcome has occurred enables clinical experts to search for a potential special cause more effectively. In this paper, we develop change point estimation methods for survival time of a clinical procedure in the presence of patient mix in a Bayesian framework. We apply Bayesian hierarchical models to formulate the change point where there exists a step change in the mean survival time of patients who underwent cardiac surgery. The data are right censored since the monitoring is conducted over a limited follow-up period. We capture the effect of risk factors prior to the surgery using a Weibull accelerated failure time regression model. Markov Chain Monte Carlo is used to obtain posterior distributions of the change point parameters including location and magnitude of changes and also corresponding probabilistic intervals and inferences. The performance of the Bayesian estimator is investigated through simulations and the result shows that precise estimates can be obtained when they are used in conjunction with the risk-adjusted survival time CUSUM control charts for different magnitude scenarios. The proposed estimator shows a better performance where a longer follow-up period, censoring time, is applied. In comparison with the alternative built-in CUSUM estimator, more accurate and precise estimates are obtained by the Bayesian estimator. These superiorities are enhanced when probability quantification, flexibility and generalizability of the Bayesian change point detection model are also considered.

## Introduction

A control chart monitors the behavior of a process over time by taking into account the stability and dispersion of the process. The chart signals when a significant change has occurred. This signal can then be investigated to identify potential causes of the change and corrective or preventive actions can then be conducted. Following this cycle leads to variation reduction and process stabilization [1].

In monitoring hospital outcomes it is necessary to consider the impact of patient health on process outcomes. To this end, risk adjustment has been taken into account in the development of control charts. Steiner *et al*. [2] developed a risk-adjusted type of cumulative sum control chart (CUSUM) to monitor surgical outcomes, death, which are influenced by the state of a patient's health, age and other factors. This approach has been extended to exponential moving average control charts (EWMA) [3], [4]. Both modified procedures have been intensively reviewed and are now well established for monitoring clinical outcomes where the observations are recorded as binary data [5]–[7].

Monitoring patient survival time instead of binary outcomes of a process in the presence of patient mix has recently been proposed in the healthcare context. In this setting a continuous time-to-event variable within a follow-up period is considered. The variable may be right censored due to a finite follow-up period. Biswas and Kalbfleisch [8] developed a risk-adjusted CUSUM based on a Cox model for failure time outcomes. Sego *et al.* [9] used an accelerated failure time regression model to capture the heterogeneity among patients prior to the surgery and developed a risk-adjusted survival time CUSUM (RAST CUSUM) scheme. They showed that this procedure is more sensitive in detection of an increase in odds ratio compared to risk-adjusted CUSUM charts. Steiner and Jones [10] extended this approach by proposing an EWMA procedure based on the same survival time model discussed by Sego *et al.* [9].

The need to know the time at which a process began to vary, the so-called change point, has been raised and discussed in the context of quality control. Accurate detection of the time of change can help in the search for a potential cause more efficiently as a tighter time-frame prior to the signal in the control charts is investigated.

In a clinical study, Assareh *et al.* [11] illustrated the capabilities of change point investigation by comparing the estimated time of changes in the rate of excess use of blood products and major adverse events during and after cardiac surgery with the time of known potential causes.

A built-in change point estimator in CUSUM charts suggested by Page [12], [13] and also an equivalent estimator in EWMA charts proposed by Nishina [14] are two early change point estimators which can be applied for all discrete and continuous distributions underlying the charts. However they do not provide any statistical inferences on the obtained estimates.

In an industrial context, Samuel and Pignatiello [15] developed and applied a maximum likelihood estimator (MLE) for the change point in a process fraction nonconformity monitored by a *p-chart*, assuming that the change type is a step change. They showed how closely this new estimator detects the change point in comparison with the usual p-chart signal. Subsequently, Perry and Pignatiello [16] compared the performance of the derived MLE estimator with EWMA and CUSUM charts. These authors also constructed a confidence set based on the estimated change point which covers the true process change point with a given level of certainty using a likelihood function based on the method proposed by Box and Cox [17]. In the case of monitoring low fraction non-conforming, Noorossana *et al*. [18] derived and analyzed the MLE estimator of a step change based on the geometric distribution control chats discussed by Xie *et al*. [19].

Other methods including clustering, least squares and genetic algorithms have also been applied in the development of change point estimators for various change and process scenarios; see Amiri and Allahyari [20] for more details.

All of the estimators described above were developed assuming that the underlying distribution is stable over time. Often this assumption cannot be satisfied in monitoring clinical outcomes because the mean of the process being monitored is highly linked to individual characteristics of patients. Recently, a series of Bayesian estimators for step change [21] and linear trend [22] in odds ratios of clinical outcomes in the presence of patient mix have been proposed. These estimators were shown to be precise, highly informative and flexible for change point investigation in this context. It was also shown that the application of Bayesian hierarchical modelling (BHM) and computational methods such as Markov Chain Monte Carlo (MCMC) to change point estimation facilitates more informed inferences based on posterior distributions for the time and the magnitude of a change.

In this paper we model and detect the change point in survival time in a Bayesian framework. The change points are estimated assuming that the underlying change is a step change. In this scenario, we model the step change in the mean survival time of a clinical process. We analyze and discuss the performance of the Bayesian change point model through posterior estimates and probability based intervals. Risk-adjusted survival time CUSUM charts are reviewed in the next section. The change point model is developed and then evaluated over several change scenarios and settings. We then compare the Bayesian estimator with the CUSUM built-in estimator.

## Methods

### Risk-Adjusted Survival Time Control Charts

The survival time of a patient who has undergone cardiac surgery is affected by the rate of mortality of cardiac surgery within the hospital and also patient covariates such as age, gender, co-morbidities and so on. Risk-adjusted control charts of time-to-event are monitoring procedures designed to detect changes in a process parameter of interest, such as survival time, where the process outcomes are affected by covariates, such as risk factors. In these procedures, regression models for time are used to adjust control charts in such a way that the effects of covariates for each input, patient say, would be eliminated.

The risk-adjusted survival time CUSUM (RAST CUSUM) proposed by Sego *et al.* [9] continuously evaluates a hypothesis of an unchanged and in-control survival time distribution, 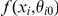, against an alternative hypothesis of a changed, out-of-control, distribution, 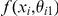 for the 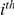 patient. In this setting the density function 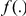 explains the observed survival time, 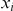, that should be adjusted based on the observed patient covariates.

The patient index 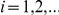 corresponds to the time order in which the patients undergo the surgery. We thus observe 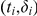 where
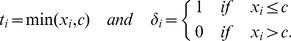
(1)


Here 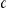 is a fixed censoring time, equal to the follow-up period. We assume that the survival time, 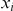, for the 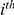 patient and consequently 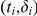, are not updated after the follow-up period. This leads to a dataset of right censored times, 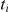.

An accelerated failure time (AFT) regression model is used to predict survival time functions, 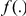, for each patient in the presence of covariates, 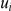. However other models such as a Cox model that also allows capture of covariates can be considered in a similar manner.

In an AFT model the survival function for the 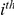 patient with covariates 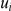, 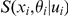, is equivalent to the baseline survival function 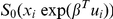, where 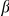 is a vector of covariate coefficients.

Several distributions can be used to model survival time with an AFT. Here we focus on the Weibull distribution and outline relevant RAST CUSUM statistics; see [23] for more details. For a Weibull distribution the baseline survival function is 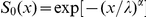 where 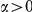 and 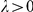 are shape and scale parameters, respectively. For the RAST CUSUM procedure, all parameters of the Weibull survival function, 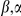 and 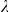, are estimated using training data, so-called phase I. In this phase, an available dataset of patients records is used assuming that the process is in-control for that period of time. A set of independent priors can also be used to obtain posterior estimates of the AFT parameters over the training data.

It has been discussed that any shifts in the quality of the process of interest can be interpreted in terms of shifts in the scale parameters, 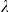; see Sego *et al.* [9] and Steiner and Jones [10]. Hence the RAST CUSUM procedure can be constructed and calibrated to detect a specific size of change in the average or median survival time (MST) since any shift in 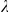 is equivalent to an identical shift in the size of average or median survival time. Thus the CUSUM score, 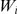, is given by
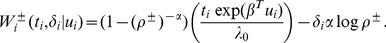
(2)where it is designed to detect an increase (a decrease) from 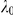 to 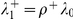 (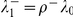). Upper and lower CUSUM statistics are obtained through 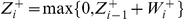 and 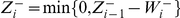, respectively, and then plotted over 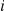. Often CUSUM statistics, 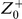 and 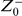, are initialized at 0.

An increase in the MST is detected when a plotted 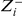 exceeds a specified decision threshold 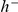; similarly, if 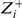 exceeds a specified decision threshold 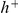, the RAST CUSUM charts signals that a decrease in the MST has occurred. Although this interpretation of a chart's signals is in contrast with the common expression used for standard risk-adjusted control charts for binary outcomes, it seems reasonable taking into account that any increase in the MST can be characterized as a drop in the odds of mortality. However in the Weibull distribution scenario for a specific change size in the MST, the equivalent magnitude of shift in odds is not obtainable; see Sego *at al*. [9] for more details.

The magnitudes of the decision thresholds in RAST CUSUM, 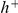 and 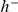, are determined in such a way that the charts have a specified performance in terms of false alarm and detection of shifts in the MST. In this regard, Markov chain and simulation approaches can be applied; see Sego [24] for more details. The proposed initialization may also be altered to achieve better performance in the detection of changes that immediately occurred after control chart construction; see Steiner [25] and Knoth [26] for more details on fast initial response (FIR).

### Change Point Model

Statistical inferences for a quantity of interest in a Bayesian framework are described as the modification of the uncertainty about their value in the light of evidence, and Bayes' theorem precisely specifies how this modification should be made as below:
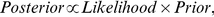
(3)where *Prior* is the state of knowledge about the quantity of interest in terms of a probability distribution before data are observed; *Likelihood* is a model underlying the observations, and *Posterior* is the state of knowledge about the quantity after the data are observed, which also is in the form of a probability distribution.

This structure may be expanded to multiple levels in a hierarchical fashion, resulting in a Bayesian hierarchical model (BHM) [27]. In complicated BHMs it is not easy to obtain the posterior distribution analytically. This analytic bottleneck has been eliminated by the the emergence of Markov chain Monte Carlo (MCMC) methods. In MCMC algorithms a Markov chain is constructed whose stationary distribution is the posterior distribution of the parameters. Samples generated from a long run of the Markov chain can then be used for posterior inferences. Some common MCMC methods include Metropolis-Hastings and the Gibbs sampler; see Robert and Casella [28] and Liu [29] for more details.

To model a change point in the presence of covariates, consider a process that results in a survival time of 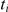, 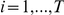, that is initially in-control. The observations can be explained by a survival function 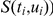, where the underlying distribution 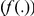 is a Weibull distribution with 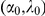, and 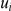 is a vectors of covariates. At an unknown point in time, 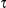, the Weibull scale parameter changes from its in-control state of 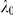 to 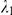, 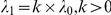 and 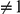. The right censored survival time step change model can thus be parameterized using survival function as follows:
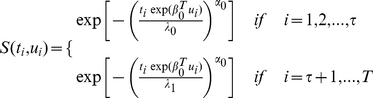
(4)where 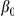 is the vector of covariate coefficients.

Assume that the process 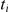 is monitored by a control chart that signals at time 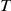.

Any probability distribution on positive values such as truncated normal, uniform and Gamma can be chosen as the prior for the magnitude of the change, 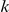, since drops, 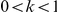 and jumps, 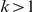, are expected in the process parameters; see Gelman *et al.* [27] for more details on selection of prior distributions. However knowing that the CUSUM control charts are very sensitive in detection of very large shifts in the process parameters and immediately signals and also the fact that the type of a detected change, either an increase or a decrease, can be distinguished by the control chart signal enable us to incorporate more information into the prior in order to gain a better performance by the proposed change point model.

In this regard, we assign a truncated normal prior distribution 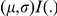 for 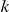 where the distribution parameters and lower and upper bounds of truncation in the indicator function 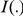 are set to correspond to the design of RAST CUSUM and the obtained signals.

For an increase in 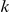 which is detected by the lower bound 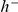 of the RAST CUSUM, we set 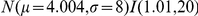. Similarly, the prior is set to 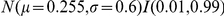 for a drop of 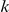 that is detected by the upper bound 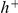 of the RAST CUSUM. This setting leads to informed priors for the magnitude of the change. The mean of both priors were set to correspond to the shifts that the chart was calibrated to detect; see Evaluation. The priors encourage sensitivity in detection of low to relatively large jumps and falls in 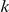.

We place a uniform distribution on the range (1, 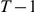) as a prior for 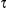 where 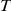 is set to the time of the signal of the control chart. See **File S1** for the step change model code in WinBUGS.

### Evaluation

We used Monte Carlo simulation to study the performance of the constructed BHM in step change detection following a signal from a RAST CUSUM control chart when a change in mean survival time is simulated to occur at 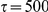. We considered the same cardiac surgery dataset that were used by Steiner *et al*. [2] and then Sego *et al*. [9] to construct risk-adjusted control charts for Bernoulli and time-to event variables, respectively. It was reported that this dataset contains 6449 operations information that were performed between 1992–1998 at a single surgical center in U.K.

The Parsonnet score [30] was recorded to quantify the patient's risk prior to the cardiac surgery. It is an additive scoring system for predicting risk in cardiac surgery based on logistic regression with 13 patient-related and three surgery-related factors [30].

A follow-up period of 30 days after the surgery was set as the censoring time. A Weibull AFT model with parameters of 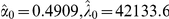 and 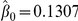 was reported by Sego *et al*. [9] when the first two years of the data were used as training data to fit the model and construct the in-control state of the process and RAST CUSWUM. They also found that the recorded Parsonnet scores of the training data can be well approximated by an exponential distribution with a mean of 8.9.

**Figure 1:**
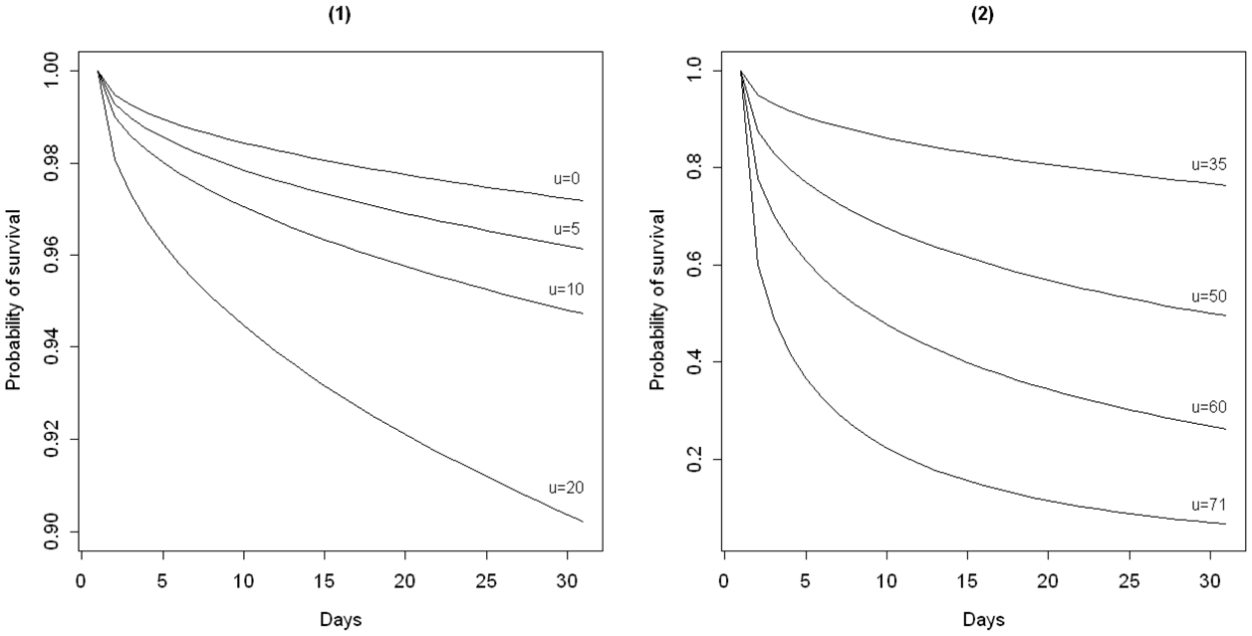
Estimated survival curves for patients over the follow-up period of 30 days. For patients with (1) low to medium and (2) medium to high Parsonnet scores (risks prior to surgery) obtained through the fitted Weibull AFT model to the training survival time data.

**Figure 2:**
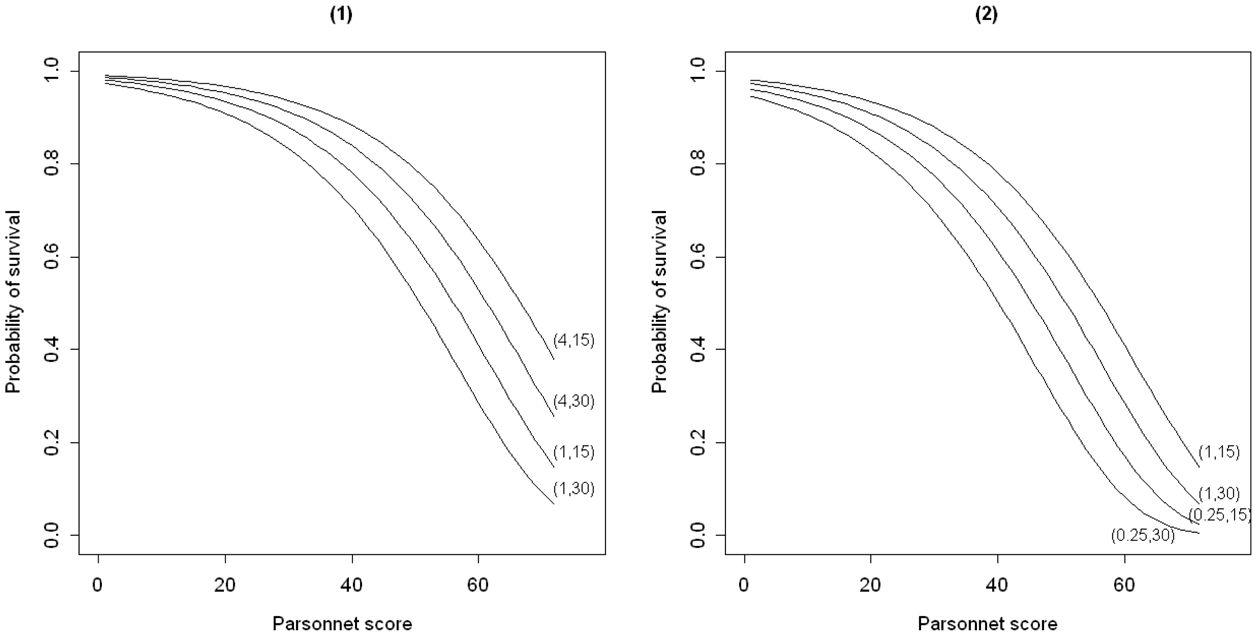
Estimated probability of survival over all Parsonnet scores prior and after changes in the MST. Probabilities at the 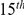 and the 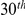 day of the follow-up period of 30 days prior and after (1) an increase of size 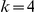, and (2) a decrease of size 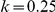 in the MST. Prior and after the change are indexed by 1 and the value of 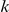.

We apply the same Weibull AFT model to simulate observations coming from the in-control state of the process. Figure 1 shows the estimated survival curves obtained through the in-control survival time model for patients with a range of different Parsonnet scores. As seen, a patient with a low score, 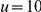 or below, is highly likely (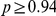) to survive within the follow-up period; see Figure 1-1. In contrast for patients with a score of 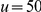 and higher, death is not unlikely within this period since the risk of death is estimated to be at least 51% for the last day shown in Figure 1-2.

To generate right censored survival time observations of a process in the in-control state 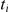, 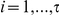, we first randomly generated the Parsonnet score, 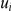, 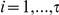, from an exponential distribution with a mean of 8.9 and then drew an associated survival time, 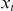, 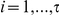, from the Weibull AFT model with 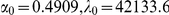, and 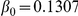. Finally, 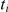 and 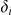 were obtained considering a censoring time of 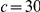 through Equation (1). Plotting the obtained observations when the associated covariates are considered results in a RAST CUSUM chart that is in-control. Note that other distributions such as uniform distributions with proper parameters or even sampling randomly from the baseline Parsonnet scores can be applied to generate covariates directly.

Because we know that the process is in-control, if an out-of-control observation was generated in the simulation of the early 500 in-control observations, it was taken as a false alarm and the simulation was restarted. However, in practice a false alarm may lead to stopping the process and analyzing root causes. When no cause is found, the process would follow without adjustment.

To generate the step change in 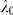, or MST, we then induced changes of sizes 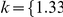, 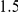, 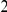, 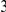, 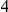, 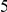, 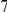, 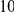, 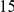, 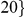 as increases and their inverse values of 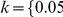, 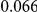, 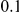, 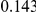, 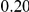, 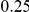, 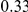, 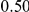, 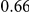, 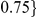 as decreases and generated observations until the control charts signalled. These changes led to different change sizes in in-control estimated survival probability over days for a patient with 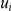 as well as survival curves between patients with different Parsonnet scores.

The effects of an increase of size 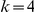 and a drop of size 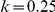 in the MST on the probability of survival at the midpoint, day 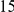, and the end, day 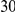, of the follow-up period for all possible Parsonnet score are demonstrated in Figure 2. As expected, the probability of survival for each patient would increase when a jump in the MST occurred. However the magnitude of this increase is larger for patients with higher Parsonnet scores.

**Figure 3:**
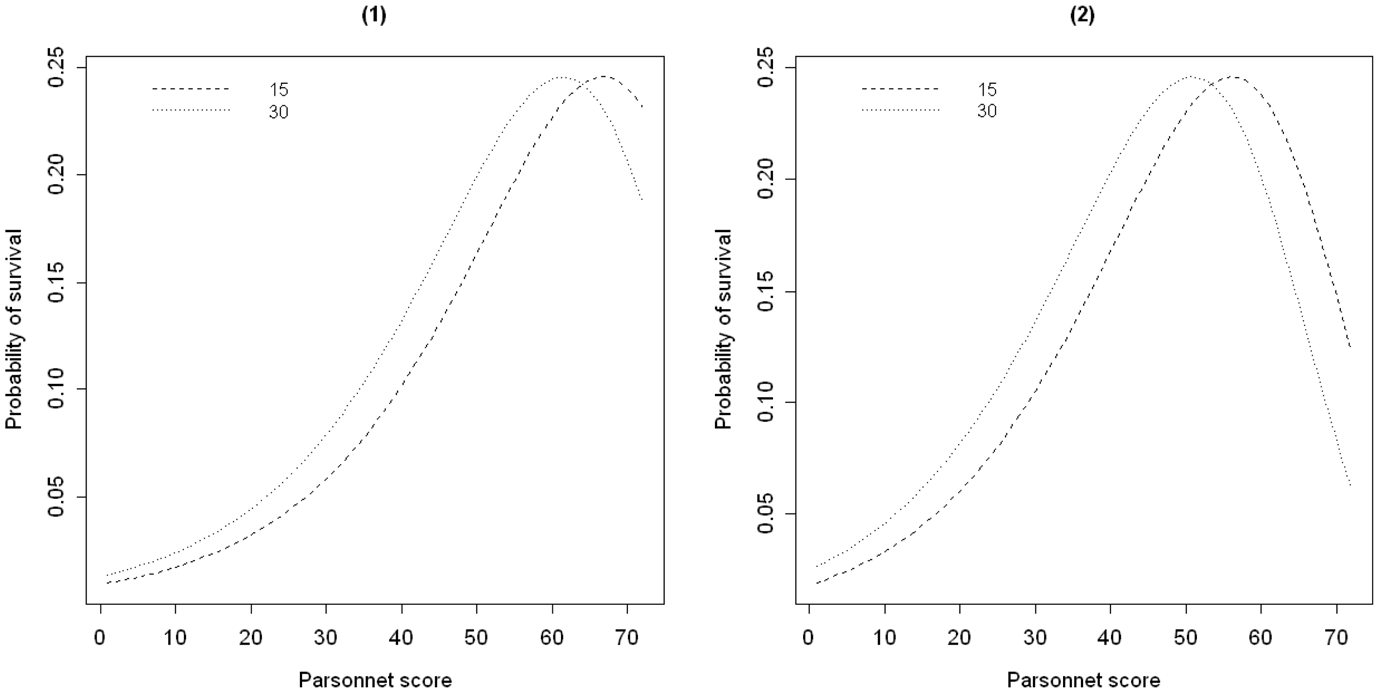
Estimated absolute magnitude of change in probability of survival over all Parsonnet scores prior and after changes in the MST. Probabilities at the 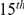 and the 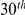 day of the follow-up period of 30 days prior and after (1) an increase of size 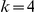, and (2) a decrease of size 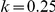 in the MST.

It was also found that the resultant magnitude of the shift in the probability of survival for an individual patient with a covariate of 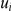, is not constant over days. The magnitude of increases in the probability at the end of period are slightly higher than those obtained for the midpoint of the period caused by a jump of 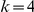 in the MST for patients with Parsonnet scores of less than 63; compare absolute change in probability for the days 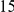 and 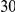 of the follow-up period before (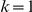) and after the increase (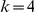) in Figure 3-1. As shown for patients with higher scores, the increase in probability for the end of the follow-up period is less than the midpoint. The same behavior was also observed for a drop of size 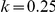; however the superiority of resultant magnitude of the shift in the probability for the end of the period tends to decline and underlie the corresponding probability for the midpoint of the period over a wider range of Parsonnet scores; see Figure 3-2.

To construct a RAST CUSUM, we applied the procedures outlined in control chart section. We calibrated the RAST CUSUM to detect an increase and a decrease in the MST that correspond to a halving and a doubling of the odds ratio within the follow-up period and with an in-control average run length (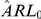) of approximately 10000 observations. As noted before, for the Weibull AFT model the corresponding odds ratio formula, discussed by Sego *et al*. [9], is not reduced to a closed form of 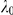 and 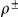 since the covariate term is not simplified in
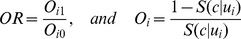
(5)where 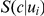 is the probability of survival at the end of follow-up period, 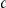.

Therefore we used Monte Carlo simulation to estimate the corresponding 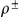. To do so, we set 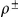 such that over 100,000 replications of generating Parsonnet scores from the fitted exponential distribution with a mean of 8.9 and calculating associated probability of survival at the end of the follow-up period of 30 days using Equation (4), and then constructing the odds in Equation (5), the desired odds ratios of size 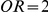 and 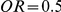 were obtained on average. An increase of 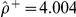 and a decrease of 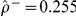 in the MST were found to correspond to the desired drop and jump in odds ratio, respectively.

We also used Monte Carlo simulation to determine decision intervals, 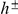. However other approaches may also be considered; see Steiner *et al*. [2] and Sego *et al*. [9]. This setting led to decision intervals of 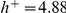 and 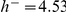. As two sided charts were considered, the negative value of 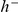 was used. The associated CUSUM scores were also obtained through Equation (2) considering the generated 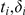 and 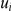.

The step change and the control chart were simulated in the statistical package R version 2.12.2 (http://www.r-project.org). To obtain posterior distributions of the time and the magnitude of the changes for each change point scenario, we used the R2WinBUGS interface [31] to generate 100,000 MCMC samples in WinBUGS version 1.4.3 [32], with the first 20000 samples ignored as burn-in. This package employs standard MCMC algorithms such as Gibbs, Metropolis and slice sampling, depending on the nature of the conditional distributions, to draw the MCMC samples; see the documentation associated with the software for further details. The posteriors were obtained in 14 minutes in average using a machine equipped by a CPU Intel dual core 2.53 GHz and 3GB RAM. We then analyzed the results using the coda package in R [33]. See **File S1** for the step change model code in WinBUGS.

## Results and Discussion

### Performance Analysis

**Figure 4:**
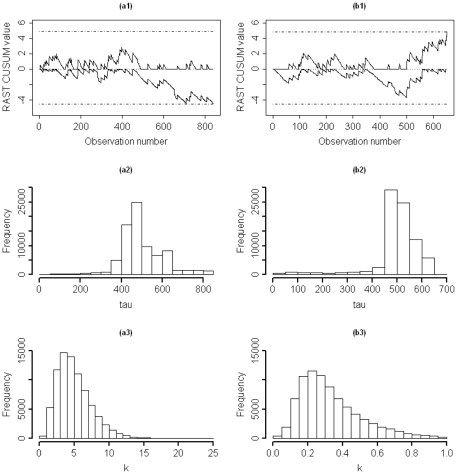
Risk-adjusted survival time CUSUM charts ( 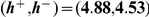
 **) and obtained posterior distributions following changes in the MST.** The RAST CUSUM chart and posteriors of the time 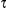 and the magnitude 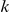 of (a1–a3) an increase of size 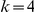, and (b1–b3) a decrease of size 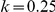 in 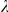 (mean survival time) where 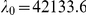 and 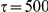.

To demonstrate the results of Bayesian change point detection in risk-adjusted control charts, we induced a jump and a drop of sizes 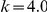 and 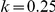, respectively, at time 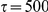 in an in-control process with an overall survival time of 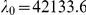. The RAST CUSUM chart detected the changes and signalled at the 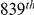 and 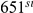 observations, corresponding to delays of 339 and 151 observations as shown in Figures 4-a1 and 4-b1, respectively. The posterior distributions of time and magnitude of the change were then obtained using MCMC discussed in Evaluation. Both distributions of the time of the change, 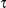, concentrate on the 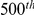 observation, approximately, as seen in Figures 4-a2 and 4-b2. The posterior for the magnitude of the change, 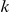, also reasonably identified the exact change sizes as it highly concentrates on values of around 4.0 and 0.25 shown in Figure 4-a3 and 4-b3.

This investigation was replicated using a smaller shift in both direction, 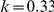 and 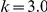 in 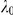. Table 1 summarizes the posterior estimates for all scenarios. If the posterior was asymmetric and skewed, the mode of the posterior was used as an estimator for the change point model parameters (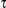 and 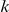). The results imply that although the obtained posterior underestimated the change point, except for 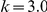, they still performed substantially better than the RAST CUSUM charts.

**Table 1:** Posterior estimates (mode, sd.) of step change point model parameters (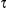 and 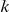) following signals (RL) from RAST CUSUM (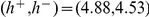) where 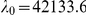 and 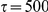.

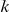	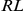	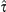	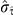	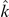	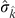
0.25	651	499.8	96.0	0.226	0.18
0.33	722	494.8	160.6	0.27	0.19
3.00	1107	734.1	165.8	3.52	3.6
4.00	839	496.3	109.1	3.68	2.4

Applying the Bayesian framework enables us to construct probability based intervals around estimated parameters. A credible interval (CI) is a posterior probability based interval which involves those values of highest probability in the posterior density of the parameter of interest. Table 2 presents 50% and 80% credible intervals for the estimated time and the magnitude of changes in 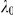 for the RAST CUSUM chart. As expected, the CIs are affected by the dispersion and higher order behaviour of the posterior distributions. Under the same probability of 0.5, the CI for the time of the change of size 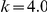 covers only eight obsrevations around the 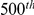 observation whereas it increases to 210 observations for 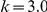 due to the larger standard deviation; see Table 1. In this scenario, the true change point does not exist in both CIs whereas the intervals obtained for the equavalent change size in the opposite direction, 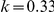, are highly informative.

**Table 2:** Credible intervals for step change point model parameters (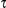 and 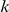) following signals (RL) from RAST CUSUM (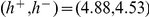) where 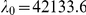 and 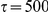.

2* 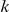	CI 50%		CI 80%	
	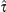	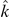	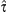	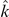
0.25	(488, 551)	(0.14, 0.33)	(453, 581)	(0.09, 0.48)
0.33	(487, 648)	(0.15, 0.40)	(359, 709)	(0.09, 0.57)
3.00	(681, 891)	(2.08, 5.74)	(604, 995)	(1.52, 9.32)
4.00	(397, 505)	(2.48, 5.39)	(389, 611)	(1.51, 7.20)

This investigation can be extended to other shift sizes for the time estimates. As shown in Table 1 and discussed above, the magnitudes of the changes are also estimated reasonably well and Table 2 shows that in all cases the real sizes of the changes are contained in the respective posterior 50% and 80% CIs.

Having a distribution for the time of the change enables us to make other probabilistic inferences. As an example, Table 3 shows the probability of the occurrence of the change point in the last {25, 50, 100, 200, 300, 400, 500} observations prior to signalling in the control charts. For a step change of size 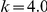 in the mean survival time, since the RAST CUSUM signals late (see Table 1), it is unlikely that the change point occurred in the last 200 observations. A considerable growth in the probability is seen when the next 200 observations are included, reaching to 0.72, whereas for a smaller increase of size 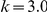, it is still not unlikely that the change point has occurred prior the last 400 observations with a probability of 0.43. For drops, 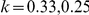, the likelihood of occurrence of the change in the last 200 observations are noticeably high since more precise posteriors of time were obtained; see Table 1.

**Table 3:** Probability of the occurrence of the change point in the last {25, 50, 100, 200, 300, 400, 500} observations prior to signalling for RAST CUSUM (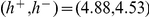) where 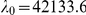 and 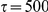.

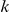	25	50	100	200	300	400	500
0.25	0.03	0.07	0.20	0.89	0.94	0.96	0.97
0.33	0.01	0.05	0.20	0.59	0.77	0.82	0.90
3.0	0.00	0.01	0.02	0.12	0.40	0.57	0.82
4.0	0.01	0.02	0.04	0.08	0.27	0.72	0.96

The above studies were based on a single sample drawn from the underlying distribution. To investigate the behavior of the Bayesian estimator over different sample datasets, for different changes in 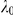, we replicated the simulation method explained in Evaluation 100 times. This replication allows us to have distribution of estimates with standard errors of the order of 10. The number of replications is a compromise between computational time, posterior estimation and particular tail probabilities. Table 4 shows the average of the estimated parameters obtained from the replicated datasets where there exists a step change in 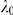 of size 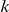. Using Monte Carlo simulation an equivalent odds ratio of mortality in the follow-up period, 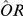, for each step change in the MST was also obtained.

**Table 4:** Average of posterior estimates (mode, sd.) of step change point model parameters (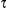 and 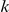) for a change in the mean survival time following signals (RL) from RAST CUSUM (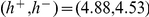) where 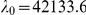 and 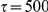.

			Change point		Change size	
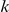	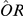	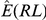	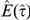	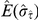	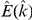	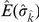
0.05	4.73	542.4	486.0	91.2	0.077	0.173
		(16.2)	(57.3)	(34.7)	(0.086)	(0.022)
0.066	3.94	554.8	490.5	92.9	0.083	0.177
		(26.6)	(62.5)	(36.7)	(0.075)	(0.025)
0.10	3.26	568.3	485.7	99.4	0.127	0.181
		(39.7)	(70.9)	(33.9)	(0.094)	(0.017)
0.143	2.70	594.2	487.3	110.9	0.154	0.182
		(49.2)	(72.5)	(34.5)	(0.090)	(0.016)
0.20	2.26	624.7	503.7	119.5	0.182	0.183
		(71.3)	(87.1)	(36.6)	(0.103)	(0.018)
0.25	2.02	692.3	527.3	132.9	0.231	0.183
		(150.4)	(146.2)	(53.4)	(0.111)	(0.018)
0.33	1.75	779.6	554.3	153.9	0.27	0.186
		(187.7)	(162.3)	(58.9)	(0.118)	(0.023)
0.50	1.41	1139.0	661.8	258.9	0.43	0.188
		(605.0)	(287.7)	(173.0)	(0.16)	(0.028)
0.66	1.23	2369.4	1270.3	562.1	0.57	0.193
		(1169.8)	(783.2)	(456.6)	(0.22)	(0.047)
0.75	1.16	2773.4	1748.0	697.9	0.63	0.195
		(2195.4)	(1304.4)	(720.8)	(0.25)	(0.047)
1.33	0.87	2921.9	2080.6	635.8	1.59	3.25
		(2629.8)	(1674.0)	(763.4)	(2.57)	(0.747)
1.5	0.81	2438.8	1764.9	510.0	1.85	3.60
		(1671.8)	(1238.9)	(555.5)	(2.59)	(0.788)
2.0	0.70	1454.0	928.8	291.9	2.69	3.81
		(626.9)	(434.4)	(197.6)	(2.10)	(0.819)
3.0	0.58	1004.7	645.1	179.9	3.60	3.98
		(382.2)	(250.3)	(98.8)	(2.19)	(0.618)
4.0	0.50	828.8	525.9	137.0	4.12	4.08
		(196.5)	(134.9)	(68.0)	(2.26)	(0.401)
5.0	0.45	785.6	514.5	113.7	5.79	4.14
		(170.2)	(128.8)	(63.5)	(2.42)	(0.394)
7.0	0.38	753.2	493.1	106.1	6.69	4.17
		(125.4)	(100.9)	(46.8)	(2.36)	(0.364)
10.0	0.32	692.4	471.8	95.3	8.90	4.27
		(89.6)	(90.9)	(43.2)	(2.20)	(0.291)
15.0	0.26	689.5	467.6	88.7	12.25	4.38
		(84.7)	(78.2)	(41.6)	(2.23)	(0.270)
20.0	0.22	670.7	465.2	80.1	14.73	4.45
		(61.6)	(73.5)	(35.3)	(2.04)	(0.148)

Standard deviations are shown in parentheses.

As seen, the RAST CUSUM control chart tends to detect larger shifts in the MST with less delays. The chart tends to fail in detection of small jumps since signals with a long delay of more than 954 observations were obtained when the MST doubled, 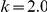. This behavior is also consistent over small drops. Having said that, more accuracy and precision are associated with the RAST CUSUM signals over drops in MST. This superiority can be explained by the nature of censored data. Since survival time are right censored, the effects of improvements is less observable and detectable than deteriorations. In other words, the data obtained after an increase in the MST is less informative than those obtained following a drop; see the next section.

For a large jump in the MST, 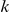 of size 7.0 or more, the average values of the modes, 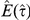, tends to underestimate the time of the change since it reports at best the 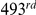 observation for 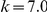. However, the Bayesian estimator still outperforms the chart signal with less bias over large increases. For inverse change sizes, large falls, the posterior mode also reports the true change point with less bias than the chart's signal. The magnitude of this bias is less than those obtained over jumps in the MST (drops in the odds ratio). The superiority of the Bayesian estimator over the chart's signal also persists for moderate shifts in the MST.

Table 4 shows that the Bayesian estimator of time, 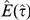, tends to overestimate the time of the change over moderate to small step changes. This bias dramatically increases over small to very small shifts, a drop of size 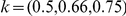 and their inverse values for jumps, reaching to a bias of 1080 observations obtained for 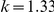, yet significantly outperforms the chart's signal. However, it may still be considered as an informative estimate of the time of the change.

Table 4 indicates that the average of the Bayesian estimator of the magnitude of the change, 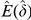, identifies change sizes with some bias. For large drops, this estimator tends to overestimate the change size whereas it underestimates the size over moderate to small drops. This estimator behaves conversely over jumps. Having said that, Bayesian estimates of the magnitude of the change must be studied in conjunction with their corresponding standard deviations. In this manner, analysis of credible intervals is effective.

### The Effect of Censoring Time

Specification of the time 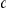 at which the survival times are right censored, affects the resulting performance of the RAST CUSUM chart. Sego *et al*. [9] have addressed construction of a RAST CUSUM chart in an updating fashion that uses longer censoring times. Here we investigated the performance of the chart and the proposed Bayesian estimator of the change point over longer censoring times. Using the simulation procedure discussed in Evaluation and followed above, we replicated generating in-control and out-of-control states of the sample process and change point detections for a selection of decreases, 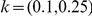, and increases, 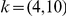, in the MST where the observed survival times are right censored using follow-up periods of 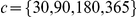 which correspond to, a month, a quarter, a half and a full year, respectively. Note that the RAST CUSUM chart was not re-calibrated based on the new censoring time since it was assumed that no updates were obtained for the patients in the training dataset.

Table 5 shows that when a longer censoring time is used, the chart detects a fall with a less delay. For a large reduction of size 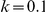 this delay drops by 26 observations on average when a follow-up period of 90 days is considered instead of the common 30 days. In this scenario, applying longer periods improves the run length since more accurate and precise 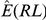 were obtained. However it is not as significant as that observed by replacing a month with a quarter of a year. This behavior is consistent over a moderate drop of size 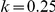. The average of the Bayesian estimator of the time, 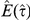, also shows that estimates with less biases and variations would be obtained if a longer follow-up period was used.

**Table 5:** Average of posterior estimates (mode, sd.) of step change point model parameters (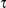 and 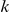) for a change in the mean survival time using different censoring time, 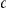, following signals (RL) from RAST CUSUM (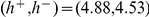) where 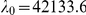 and 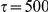.

	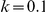			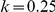			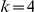			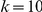		
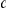	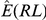	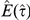	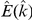	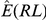	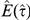	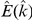	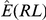	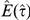	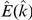	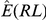	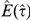	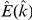
30	568.3	485.7	0.127	692.3	527.3	0.231	828.8	525.9	4.12	692.4	471.8	8.90
	(39.7)	(70.9)	(0.094)	(150.4)	(146.2)	(0.111)	(196.5)	(134.9)	(2.26)	(89.6)	(90.9)	(2.20)
90	542.8	486.3	0.124	609.9	513.7	0.233	708.5	517.3	4.38	621.3	473.3	8.81
	(19.5)	(62.3)	(0.102)	(77.6)	(63.0)	(0.119)	(126.6)	(115.9)	(2.38)	(55.9)	(79.5)	(2.40)
180	534.8	492.6	0.124	579.4	509.7	0.213	645.6	515.2	4.16	591.4	488.2	8.72
	(16.1)	(36.5)	(0.101)	(46.5)	(50.6)	(0.126)	(97.3)	(86.2)	(2.41)	(37.9)	(40.4)	(2.56)
365	527.9	489.7	0.148	562.6	495.3	0.227	604.7	512.2	4.29	562.7	489.1	8.92
	(12.5)	(33.6)	(0.139)	(44.4)	(63.9)	(0.130)	(69.7)	(76.2)	(2.52)	(25.4)	(32.4)	(2.71)

Standard deviations are shown in parentheses.

The behavior of the chart and the estimator observed for drops persists over increases in the MST as well. Having said that, it seems to be more significant over increases. The Bayesian estimator of the time, 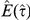, also tends to detect the change point more accurately and precisely since less overestimation and underestimation were observed over a longer censoring time for moderate and large jumps, respectively.

Although the discussed results are in favor of following up patients for a longer time, care should be taken in this approach since the possibility of contribution of other risk factors rather than the process of interest, cardiac surgery, in the observed survival time increases. Investigation of incorporating such post-surgery factors and also the effect of re-calibration of the RAST CUSUM is left for further research.

### Comparison of Bayesian Estimator with Other Methods

To study the performance of the proposed Bayesian estimators in comparison with that introduced in Introduction, we run the available alternative, built-in estimator of the CUSUM chart, within the replications discussed in Performance Analysis.

Based on the suggestion by Page [12], if an increase in a process rate is detected by CUSUM charts, an estimate of the change point is obtained through 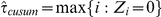. Similarly for detection of a decrease, the estimated change point is 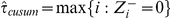.

Table 6 shows the average of the Bayesian estimates, 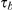, and detected change points provided by the built-in estimator of CUSUM, 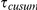 charts for shifts in the mean survival time, 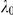 say.

**Table 6:** Average of detected time of a step change in the mean survival time obtained by the Bayesian estimator (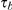) and CUSUM built-in estimator following signals (RL) from RACUSUM (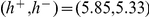) where 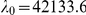 and 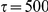.

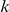	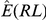	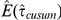	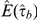
0.05	542.4	458.22	486.0
	(16.2)	(77.9)	(57.3)
0.066	554.8	467.5	490.5
	(26.6)	(80.9)	(62.5)
0.10	568.3	456.0	485.7
	(39.7)	(79.9)	(70.9)
0.143	594.2	474.5	487.3
	(49.2)	(67.1)	(72.5)
0.20	624.7	477.1	503.7
	(71.3)	(75.6)	(87.1)
0.25	692.3	523.5	527.3
	(150.4)	(138.4)	(146.2)
0.33	779.6	565.4	554.3
	(187.7)	(158.9)	(162.3)
0.50	1139.0	903.7	661.8
	(605.0)	(568.0)	(287.7)
0.66	2369.4	2289.5	1270.3
	(1169.8)	(1168.2)	(783.2)
0.75	2773.4	2426.0	1748.0
	(2195.4)	(1906.9)	(1304.4)
1.33	2921.9	2561.2	2080.6
	(2629.8)	(2655.8)	(1674.0)
1.5	2438.8	2035	1764.9
	(1671.8)	(1672.0)	(1238.9)
2.0	1454.0	997.6	928.8
	(626.9)	(597.2)	(434.4)
3.0	1004.7	635.1	645.1
	(382.2)	(332.1)	(250.3)
4.0	828.8	468.4	525.9
	(196.5)	(174.0)	(134.9)
5.0	785.6	470.0	514.5
	(170.2)	(160.6)	(128.8)
7.0	753.2	455.2	493.1
	(125.4)	(100.5)	(100.9)
10.0	692.4	417.0	471.8
	(89.6)	(122.2)	(90.9)
15.0	689.5	432.3	467.6
	(84.7)	(102.4)	(78.2)
20.0	670.7	430.5	465.2
	(61.6)	(112.9)	(73.5)

Standard deviations are shown in parentheses.

The built-in estimator of CUSUM charts outperforms associated signals over all shifts in the MST; however it tends to significantly underestimate the exact change point when the magnitude of the shifts increases. It has been previously discussed that the RAST CUSUM has a better performance over drops; this finding persists for the built-in estimator since less bias and higher precision are associated with the change point estimates over drops. Having said that, the superiority of the built-in estimator over the chart's signal is more significant over jumps in the MST.

Although the Bayesian estimator, 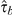, tends to underestimate the time of changes over large shifts, 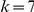 or more, and their inverse, it outperforms the built-in estimator, 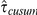, with less bias reaching to 15 and 35 observations over large drops and jumps, respectively.

The posterior mode tends to overestimate the true change point over moderate to small shift sizes, yet it reports more accurate results than the alternative. In the only exceptional scenarios, a shift of sizes 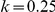 and 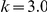, where less bias is associated with the built-in estimator, no significant superiority is gained when the obtained variation of the estimates is also taken into account. Comparison of variation of estimated change points across other scenarios of shifts in the mean survival time also supports the superiority of the Bayesian estimator over the alternative.

### Conclusion

Obtaining accurate information about the time when a change occurred in the process has been recently considered within quality control applications. Indeed, knowing the change point enhances efficiency of root cause analysis efforts by restricting the search to a tighter window of observations and related variables.

In this paper, using a Bayesian framework, we modeled change point estimation in time-to-event data for a clinical process with dichotomous outcomes, death and survival, where patient mix was present. We considered a range of jumps and falls in the mean survival time of an in-control process. We constructed Bayesian hierarchical models and derived posterior distributions for change point estimates using MCMC. The performance of the Bayesian estimators was investigated through simulation in conjunction with RAST CUSUM control charts for monitoring right censored survival time of patients who underwent cardiac surgery procedures within a follow-up period of 30 days. Here the severity of risk factors prior to the surgery was evaluated by the Parsonnet score.

The results showed that the Bayesian estimates significantly outperform the RAST CUSUM control charts in change detection over different magnitudes of shifts in the mean survival time. These results highlight that post-signal change point investigation can enhance the efficiency of root cause analysis efforts in monitoring time-to-event outcomes. It was also shown that over longer follow-up periods better estimates were provided by the RAST CUSUM chart and the Bayesian estimator. However, care should be taken in practice since the effects of unseen contributors in the observed survival model may increase over longer follow-up periods. The comparison of the Bayesian estimator with built-in estimators of CUSUM revealed that the Bayesian estimator performed reasonably well and outperformed the alternatives.

In addition to the accuracy and precision criteria used for the comparison study, the posterior distributions for the time and the magnitude of a change enable us to construct probabilistic intervals around estimates and probabilistic inferences about the location of the change point. This is a significant advantage of the proposed Bayesian approach over other methods that produce only MLE estimators. Furthermore, the flexibility of the Bayesian hierarchical models, ease of extension to more complicated change scenarios such as linear and nonlinear trends in survival time, relief from analytic calculation of the likelihood function through MCMC, and ease of coding with available packages should be considered as additional benefits of the proposed approach. The only drawback of the Bayesian approach is that it is more time-consuming; however this was not particularly onerous in the studies presented here.

The investigation conducted in this study was based on a specific in-control rate of mortality observed in the pilot hospital. Although it is expected that superiority of the proposed Bayesian estimator persists over other processes in which the in-control rate and the distribution of baseline risk may differ, the results obtained for estimators and control charts over various change scenarios motivates replication of the study using other patient mix profiles and underlying models. Moreover modification of change point model elements such as replacing priors with non-informative and informative alternatives may be of interest.

## Supporting Information

File S1 **WinBUGS code for change point model.**
(TXT)
